# Compulsive sexual behavior: Prefrontal and limbic volume and interactions

**DOI:** 10.1002/hbm.23447

**Published:** 2016-10-27

**Authors:** Casper Schmidt, Laurel S. Morris, Timo L. Kvamme, Paula Hall, Thaddeus Birchard, Valerie Voon

**Affiliations:** ^1^ Department of Psychiatry University of Cambridge, Herchel Smith Buidling for Brain & Mind Sciences Forvie Site, Robinson Way Cambridge CB2 0SZ United Kingdom; ^2^ Cognitive Neuroscience Research Unit, Danish Neuroscience Center Aarhus University Hospital Nørrebrogade 44, building 10G 8000 Aarhus C Denmark; ^3^ Center of Functionally Integrative Neuroscience/MINDLab Danish Neuroscience Center, Aarhus University Hospital Nørrebrogade 44, building 10G, 8000 Aarhus C Denmark; ^4^ Behavioral and Clinical Neurosciences Institute, Department of Psychology University of Cambridge Downing Street Cambridge CB2, 3EB United Kingdom; ^5^ United Kingdom Council for Psychotherapy 2nd Floor, Edward House 2 Wakley Street London EC1V 7LT United Kingdom; ^6^ Cambridgeshire and Peterborough NHS Foundation Trust Cambridge Rd, Fulbourn Cambridge CB21 5HH United Kingdom

**Keywords:** compulsive sexual behavior, sexual addiction, voxel‐based morphometry, resting state functional MRI, depression

## Abstract

**Background:**

Compulsive sexual behaviors (CSB) are relatively common and associated with significant personal and social dysfunction. The underlying neurobiology is still poorly understood. The present study examines brain volumes and resting state functional connectivity in CSB compared with matched healthy volunteers (HV).

**Methods:**

Structural MRI (MPRAGE) data were collected in 92 subjects (23 CSB males and 69 age‐matched male HV) and analyzed using voxel‐based morphometry. Resting state functional MRI data using multi‐echo planar sequence and independent components analysis (ME‐ICA) were collected in 68 subjects (23 CSB subjects and 45 age‐matched HV).

**Results:**

CSB subjects showed greater left amygdala gray matter volumes (small volume corrected, Bonferroni adjusted *P* < 0.01) and reduced resting state functional connectivity between the left amygdala seed and bilateral dorsolateral prefrontal cortex (whole brain, cluster corrected FWE *P* < 0.05) compared with HV.

**Conclusions:**

CSB is associated with elevated volumes in limbic regions relevant to motivational salience and emotion processing, and impaired functional connectivity between prefrontal control regulatory and limbic regions. Future studies should aim to assess longitudinal measures to investigate whether these findings are risk factors that predate the onset of the behaviors or are consequences of the behaviors. *Hum Brain Mapp 38:1182–1190, 2017*. © 2016 Wiley Periodicals, Inc.

**Abbreviations**
ACC: anterior cingulate cortex
CSB: compulsive sexual behavior
CSF: cerebrospinal fluid
DLPFC: dorsolateral prefrontal cortex
GM: gray matter
GLM: general linear model
HV: healthy volunteers
MPRAGE: magnetization prepared gradient‐echo
OFC: orbitofrontal cortex
ROI: region of interest
SPM: Statistical Parametric Mapping
TR: repetition time
TE: echo time
VBM: voxel‐based morphometry
WM: white matter.

## INTRODUCTION

Compulsive sexual behavior (CSB), also known as hypersexual disorder or sexual addiction, is relatively common (estimated at 3%–6%) [Kraus et al., 2016] and associated with significant distress and psychosocial impairments, including being characterized by craving, impulsivity, and social and occupational impairment [Kraus et al., 2016]. Recent studies have focused on understanding underlying neurobiological correlates [Kraus et al., 2016] although the paucity of studies limits our understanding of underlying mechanisms and how we might conceptualize these disorders. CSB has been conceptualized as either an impulse control disorder or a behavioral addiction [Kraus et al., 2016]. However, although criteria for hypersexual disorder were proposed for the DSM‐5 and validated in the field trial [Reid et al., 2012], this disorder along with the pathological use of the internet or video games, were not included in the main section of the DSM‐5, in part due to limited data on the conditions. Thus, further studies on CSB are necessary to develop a greater understanding of these disorders. Although CSB can have a range of behaviors, here we focus on a group reporting predominant difficulties with compulsive pornography use. We have used the term CSB on the assumption that “compulsive” describes the repetitive phenomenology and is not intended to imply any mechanistic or etiological assumptions.

We conducted a review of the literature on behavioral addictions using either voxel‐based morphometry (VBM) or cortical thickness. We used the following search words on PubMed (http://www.ncbi.nlm.nih.gov/pubmed): ‘[(“voxel‐based morphometry” or “cortical thickness”) and],' followed by either “[pathological gambling],” “[internet addiction],” “[internet disorder],” “[video gaming addiction],” or “[gaming addiction].” In total, 13 studies were found within behavioral addictions related to gambling, internet use, or video gaming that assessed either VBM or cortical thickness. The review of the literature is presented in Table 1 and discussed below.

**Table 1:** Literature review of volumetric and cortical thickness studies on behavioral addictions

Title	Behavioral addiction	Subjects (P/HV)	Measure	Regions implicated
[Grant et al., 2015]	Gambling disorder	16/17	Cortical thickness	Decreased cortical thickness in r‐SFC, RMFC, MOFC, PCG and bl‐IPC
[Joutsa et al., 2011]	Pathological gambling	12/12	Voxel‐based morphometry	No volumetric differences in GM or WM between HV and patients
[Koehler et al., 2013]	Pathological gambling	20/21	Voxel‐based morphometry	Increased GM volume in bl‐VS and r‐PFC
[van Holst et al., 2012]	Problem gambling	40/54	Voxel‐based morphometry	No volumetric differences in GM or WM between problem gamblers and HV
[Hong et al., 2013]	Internet addiction	15/15	Cortical thickness	Decreased cortical thickness in r‐LOFC
[Yuan et al., 2011]	Internet addiction	18/18	Voxel‐based morphometry	Decreased GM volume in DLPFC, SMA, OFC, CB, RACC
[Zhou et al., 2011]	Internet addiction	15/18	Voxel‐based morphometry	Decreased GM density in l‐ACC, PCC, IC, LING
[Lin et al., 2014]	Internet gaming addiction	35/36	Voxel‐based morphometry	Decreased GM density in the IFG, l‐CG, IC, and r‐HIPP Decreased WM density in IFG, IC, IPC, ACC
[Sun et al., 2014]	Internet gaming addiction	18/21	Voxel‐based morphometry	Increased GM volume in the r‐ITG, MTG, PHG Decreased GM volume in l‐PrCG
[Wang et al., 2015]	Internet gaming disorder	28/28	Voxel‐based morphometry	Decreased GM volume in ACC, PCUN, SMA, SPC, and l‐DLPFC, IC, CB
[Cai et al., 2015]	Internet gaming disorder	27/30	Subcortical volume, FreeSurfer	Increased volumes of CN and VS
[Weng et al., 2013]	Online game addiction	17/17	Voxel‐based morphometry	Decreased GM volume in r‐OFC, SMA and bl‐IC
[Yuan et al., 2013]	Online game addiction	18/18	Cortical thickness	Increased cortical thickness in l‐PrCG, PCUN, MFC, ITG, MTG Decreased cortical thickness in l‐LOFC, IC, r‐PCG, IPC

Abbreviations: HV, healthy volunteers; P, patients; r, right; l, left; bl, bilateral; GM, gray matter; WM, white matter; ACC, anterior cingulate cortex; CB, cerebellum; CG, cingulate gyrus; CN, caudate nucleus; DLPFC, dorsolateral prefrontal cortex; HIPP, hippocampus; IC, insular cortex; IFG, inferior frontal gyrus; IPC, inferior parietal cortex; ITG, inferior temporal gyrus; LING, lingual gyrus; LOFC, lateral orbitofrontal cortex; MFC, middle frontal cortex; MOFC, medial orbitofrontal cortex; MTG, middle temporal gyrus; OFC, orbitofrontal cortex; PCC, posterior cingulate cortex; PCG, post‐central gyrus; PCUN, precuneus; PrCG, pre‐central gyrus; PFC, prefrontal cortex; PHG, parahippocampal gyrus; RACC, rostral anterior cingulate cortex; RMFC, rostral middle frontal cortex; SFC, superior frontal cortex; SMA, supplementary motor area; SPC, superior parietal cortex; VS, ventral striatum.

Insight into neural disturbances in addictions comes from studies of substance use disorders (SUD). Individuals with SUD show decreases in cortical brain volume and thickness particularly in prefrontal cortical regions that subserve flexible behavioral control. A recent meta‐analysis of 9 studies and 296 alcohol dependent individuals found significantly reduced prefrontal gray matter (GM) volumes, including anterior cingulate cortex (ACC) [Xiao et al., 2015], with frontal cortical GM volume being negatively associated with lifetime alcohol use [Taki et al., 2006]. Prefrontal GM volumes were similarly reduced in cocaine dependent individuals, including in orbitofrontal cortex (OFC) [Rando et al., 2013; Tanabe et al., 2009], anterior prefrontal cortex [Rando et al., 2013] and ACC [Connolly et al., 2013], the latter associated with years of drug use [Connolly et al., 2013].

Group differences in cortical volumes and thickness have been less clear in behavioral addictions (reviewed in Table 1). Three small studies of gambling disorder showed inconsistent findings with either decreased cortical thickness in multiple prefrontal and parietal regions [Grant et al., 2015], increased volumes in the right prefrontal cortex [Koehler et al., 2013] or no group differences [Joutsa et al., 2011]. In a large study of less severe problem gamblers, no group differences were observed in brain volumes [van Holst et al., 2012]. One small study in internet addiction showed lower cortical thickness in the OFC [Hong et al., 2013], with another reporting lower volume in dorsolateral prefrontal cortex (DLPFC) [Yuan et al., 2011] and two studies suggesting lower ACC volumes [Yuan et al., 2011; Zhou et al., 2011]. Two small studies in internet gaming disorders reported decreased volumes in OFC [Weng et al., 2013; Yuan et al., 2013], and two larger studies reported smaller volumes in cingulate cortex [Lin et al., 2014; Wang et al., 2015] with single studies reporting decreases in DLPFC [Wang et al., 2015], inferior frontal [Lin et al., 2014], superior parietal [Wang et al., 2015] and inferior parietal [Yuan et al., 2013] cortices. With respect to subcortical structures, one small study reported higher ventral striatal (VS) volumes in gambling disorder [Koehler et al., 2013] with no subcortical differences reported in the other studies. In internet gaming disorder, findings were similarly inconsistent with either greater parahippocampal [Sun et al., 2014], lower hippocampal [Lin et al., 2014] or no differences [Wang et al., 2015; Weng et al., 2013]. One study with a reasonable sample size focusing on subcortical volumes reported greater caudate and VS volumes associated with cognitive control deficits [Cai et al., 2015]. Taken together, the findings of cortical or subcortical abnormalities in gambling disorder are highly inconsistent. In contrast, reports of cortical abnormalities in internet use or internet gaming more consistently report decreased volumes with decreased ACC and OFC volume replicated across at least two studies.

To date, there is sparse evidence of structural neural changes in individuals with CSB. Studies of healthy individuals with excessive pornography use without a diagnosis of CSB show lower GM volumes in the right caudate [Kühn and Gallinat, 2014]. A small diffusion MRI study of individuals with CSB (*N* = 8 per group) showed reduced mean diffusivity in superior frontal white matter (WM) tracts compared with HV [Miner et al., 2009]. With respect to functional activity, male HV show enhanced habituation processes with lower left putaminal BOLD activity to static erotic images [Kühn and Gallinat, 2014] and lower late positive potential to explicit images [Prause et al., 2015]. In contrast, in a task‐based fMRI study comparing CSB with HV, explicit sexual videos elicited higher VS, amygdala and dorsal ACC BOLD responses in CSB [Voon et al., 2014]. Functional connectivity between these regions correlated with an index of sexual desire or “wanting” but not “liking” in CSB subjects suggesting the role of incentive motivation, paralleling substance addiction. Similarly, in another study in internet pornography addiction, the preferred sexual image was associated with greater ventral striatal activity and correlated only with self‐reported symptoms of internet pornography addiction and not with other measures of sexual behavior or depression [Brand et al., 2016]. Another recent study also shows that individuals with problematic hypersexual behavior experienced more frequent and enhanced sexual desire during exposure to sexual stimuli, and that greater activation was observed in the caudate, inferior parietal lobe, dorsal anterior cingulate gyrus, thalamus, and DLPFC in this group [Seok and Sohn, 2015]. CSB individuals further show greater early attentional bias to explicit sexual stimuli [Mechelmans et al., 2014] that correlated with choice preferences for cues conditioned to sexual images [Banca et al., 2016]. In response to repeated exposure of static erotic images, CSB subjects showed greater habituation in the dorsal ACC to sexual outcomes, which correlated with choice preferences for novel sexual images [Banca et al., 2016], an effect that may be explained by either habituation but might also be consistent with the concept of tolerance in addictions.

The current study examines volumetric GM in CSB and review the current literature on volumetric and cortical thickness studies in gambling disorder and in internet and gaming use disorders. We also examine resting state functional connectivity of individuals with CSB and matched HV with a novel multi‐echo planar sequence and independent components analysis (ME‐ICA) wherein BOLD signals are identified as independent components with linear echo time (TE)‐dependent signal change whereas non‐BOLD signals are identified as TE‐independent components [Kundu et al., 2012]. We expect a disrupted network of salience and reward‐related systems subserved by amygdala, VS and dorsal ACC.

## METHODS

### Participants

CSB subjects were recruited via internet‐based advertisements and from referrals from therapists. Age‐matched male HV were recruited from community‐based advertisements in the East Anglia area. All CSB subjects were interviewed by a psychiatrist to confirm they fulfilled diagnostic criteria for CSB (met proposed diagnostic criteria for both hypersexual disorder [Kafka, 2010; Reid et al., 2012] and sexual addiction [Carnes et al., 2007]), focusing on compulsive use of online sexually explicit material. This was assessed using a modified version of the Arizona Sexual Experiences Scale (ASES) [Mcgahuey et al., 2011], in which questions were answered on a scale of 1–8, with higher scores representing greater subjective impairment. Given the nature of the cues, all CSB subjects and HV were male and heterosexual. All HV were age‐matched (±5 years of age) with CSB subjects. Subjects were also screened for compatibility with the MRI environment as we have done previously [Banca et al., 2016; Mechelmans et al., 2014; Voon et al., 2014]. Exclusionary criteria included being under 18 years of age, having a history of SUD, being a current regular user of illicit substances (including cannabis), and having a serious psychiatric disorder, including current moderate‐severe major depression or obsessive‐compulsive disorder, or history of bipolar disorder or schizophrenia (screened using the Mini International Neuropsychiatric Inventory) [Sheehan et al., 1998]. Other compulsive or behavioral addictions were also exclusions. Subjects were assessed by a psychiatrist regarding problematic use of online gaming or social media, pathological gambling or compulsive shopping, childhood or adult attention deficit hyperactivity disorder, and binge‐eating disorder diagnosis. Subjects completed the UPPS‐P Impulsive Behavior Scale [Whiteside and Lynam, 2001] to assess impulsivity, and the Beck Depression Inventory [Beck et al., 1961] to assess depression. Two of 23 CSB subjects were taking antidepressants or had comorbid generalized anxiety disorder and social phobia (*N* = 2) or social phobia (*N* = 1) or a childhood history of ADHD (*N* = 1). Written informed consent was obtained, and the study was approved by the University of Cambridge Research Ethics Committee. Subjects were paid for their participation.

### Neuroimaging

#### Data acquisition and processing

##### Structural.

Structural images were collected including full magnetization prepared gradient‐echo (MPRAGE) using a Siemens Tim Trio 3T‐scanner with a 32‐channel head coil using a T1 weighted MPRAGE sequence (176 sagittal slices, 9 minute scans; repetition time (TR) = 2,500 ms; TE = 4.77 ms; inversion time = 1,100 ms; acquisition matrix = 256 × 256 × 176; flip angle = 7°; voxel size 1 × 1 × 1 mm). Scanning took place at The Wolfson Brain Imaging Centre at the University of Cambridge.

Structural data was processed with Statistical Parametric Mapping (SPM8; http://www.fil.ion.ucl.ac.uk/spm) (Wellcome Trust Centre for Neuroimaging, London, UK). Anatomical images were manually re‐oriented, placing the origin at the anterior commissure. Images were segmented (using New Segment for SPM) into GM, WM and cerebrospinal fluid (CSF) based on standard tissue probability maps for each tissue type. The three tissue class volumes were summed to produce estimated total intracranial volume. A custom template was created using DARTEL [Ashburner, 2007], which defines the parameters necessary to fit each individuals' native GM image to a common space, in an iterative manner. This DARTEL template was then registered to the tissue probability maps with affine transformations, bringing images into MNI space. Images were smoothed spatially with a full width at half maximum kernel of 8 mm^3^.

##### Resting state.

Resting state fMRI data were acquired for 10 minutes with eyes open with a Siemens 3T Tim Trio scanner with a 32‐channel head coil at the Wolfson Brain Imaging Centre, University of Cambridge. A multi‐echo echo planar imaging sequence was used with online reconstruction (repetition time, 2.47 s; flip angle, 78°; matrix size 64 × 64; in‐plane resolution, 3.75 mm; FOV, 240 mm; 32 oblique slices, alternating slice acquisition slice thickness 3.75 mm with 10% gap; iPAT factor, 3; bandwidth = 1,698 Hz/pixel; echo time (TE) = 12, 28, 44, and 60 ms).

Multi‐echo independent component analysis (ME‐ICAv2.5 beta6; http://afni.nimh.nih.gov) was used for analysis and de‐noising of the multi‐echo resting state fMRI data. ME‐ICA decomposes multi‐echo fMRI data into independent components with FastICA. BOLD signal percent signal change is linearly dependent on TE, a characteristic of the T2* decay. This TE‐dependence is measured using the pseudo‐*F*‐statistic, kappa, with components that scale strongly with TE having high kappa scores [Kundu et al., 2012]. Non‐BOLD components are identified by TE independence measured by the pseudo‐*F*‐statistic, rho. Components are thus categorized as BOLD or non‐BOLD based on their kappa and rho value weightings, respectively [Kundu et al., 2012]. Non‐BOLD components are removed by projection, de‐noising data for motion, physiological and scanner artifacts in a robust manner based on physical principles. Each individual's de‐noised echo planar images were coregistered to their MPRAGE and normalized to the Montreal Neurological Institute (MNI) template. Spatial smoothing was conducted with a Gaussian kernel (full width half maximum = 6 mm). The time course for each voxel was temporally band‐pass filtered (0.008 < *f* < 0.09 Hz). Each individual's anatomical scan was segmented into GM, WM, and CSF. Significant principal components of the signals from WM and CSF were removed.

Functional connectivity analysis was performed using a region of interest (ROI)‐driven approach with CONN‐fMRI Functional Connectivity toolbox [Whitfield‐Gabrieli and Nieto‐Castanon, 2012] for SPM (http://www.fil.ion.ucl.ac.uk/spm/software/spm8/).

### Statistical Analysis

Subjects' characteristics and questionnaire scores were compared between groups with two‐tailed *t*‐tests without assuming equal variance. All statistical analyses were performed using R version (3.2.0) [RC Team, 2014].

#### Structural

For group comparisons, GM volumes for CSB subjects and HV were entered into a general linear model (GLM). Data was corrected for participants' total intracranial volume using proportional scaling and an explicit mask in SPM. Group comparisons were adjusted for both age and depression scores as covariates. We focused on *a priori* hypothesized regions of interest identified in our previous study [Voon et al., 2014] and in meta‐analyses of drug cue reactivity studies [Kühn and Gallinat, 2011], namely left and right VS, left and right amygdala, and dorsal ACC using small volume corrected (SVC) family‐wise error (FWE) corrected *P* < 0.01 (Bonferroni corrected for multiple comparisons). For these SVC analyses, we used a VS anatomical ROI, previously described [Murray et al., 2008] which was hand drawn using MRIcro based on the definition of VS by Martinez et al. [2003]. The amygdala ROI was obtained from the Automated Anatomical Labelling (AAL) atlas. The dorsal ACC was manually altered using MarsBaR ROI toolbox [Brett et al., 2002] and based on the cingulate cortex ROI from the AAL atlas. It was modified such that the anterior border was the tip of the genu of the corpus callosum [Cox et al., 2014; Desikan et al., 2006] and the posterior was the posterior end of the genu of the corpus callosum [Desikan et al., 2006]. Additional analyses adjusting for BDI scores were performed.

#### Resting state

To compare connectivity between CSB subjects and HV, ROI‐to‐voxel whole brain connectivity maps were computed for the left amygdala seed region of interest based on volumetric group difference findings. Resultant connectivity maps were entered into full factorial GLM's to compare whole‐brain connectivity between groups adjusting for age with a subsequent analysis adjusting for both age and depression. Whole brain cluster corrected FWE *P* < 0.05 was considered significant for group differences.

## RESULTS

### Characteristics

Twenty‐three heterosexual men with CSB (age 26.9; SD 6.22 years) and 69 age‐matched (age 25.6; SD 6.55 years) heterosexual male HV participated in the study (Table 2), of which 19 CSB subjects and 55 HV completed behavioral questionnaires. CSB subjects had higher BDI (*P* = 0.006) and UPPS‐P (*P* < 0.001) scores compared with HV. Other behavioral scores including pattern and severity of pornography and internet use have been reported elsewhere [Mechelmans et al., 2014; Voon et al., 2014].

**Table 2:** Demographic and behavioral data for compulsive sexual behavior subjects and healthy volunteers

Group	Age	BDI	UPPS‐P
CSB (*N* = 23)	26.9 (6.22)	14.82 (11.85)^a^	152.21 (16.50)^a^
HV (*N* = 69)	25.6 (6.55)	6.03 (7.20)^b^	124.87 (20.73)^b^
*T*‐value (*P*‐value)	0.88 (*P* = 0.380)	3.04 (*P* = 0.006)	5.81 (*P* < 0.001)

Reports on standard deviations and *P*‐values for two‐sampled *t*‐tests are in brackets.

a Missing 4 participants out of 23.

b Missing 14 participants out of 69.

BDI scores of 0–13 indicate minimal depression, 14–19 mild depression, 20–28 moderate depression, and 29–63 severe depression.

UPPS‐P scores range between 59 and 236 as measures of impulsivity (59 = least impulsive; 236 = most impulsive) calculated from 59 items, each rated between 1 and 4 and representing different components of impulsivity.

Abbreviations: HV, healthy volunteers; CSB, compulsive sexual behaviors; BDI, Beck's Depression Inventory; UPPS‐P, UPPS‐P Impulsive Behavior Scale.

### Structural

**Figure 1:**
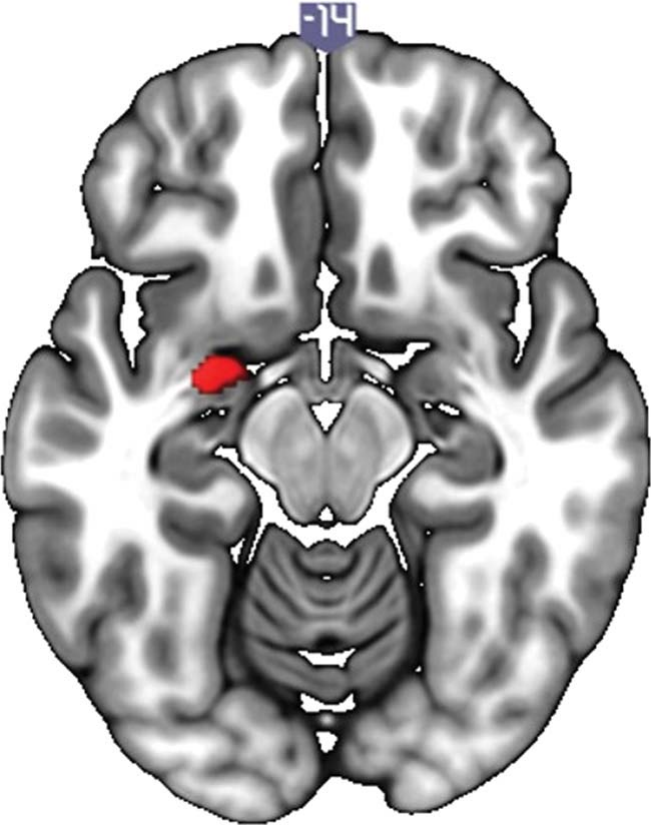
Voxel‐based morphometry in compulsive sexual behaviors. Greater left amygdala volume is shown in compulsive sexual behaviors relative to healthy volunteers. The image is thresholded at *P* < 0.005 uncorrected for illustration. [Color figure can be viewed at http://wileyonlinelibrary.com]

The ROI analyses of left and right amygdala, left and right VS and dorsal ACC revealed that left amygdala gray matter volume was increased in CSB compared with matched HV (SVC FWE‐corrected, *P* = 0.0096, *Z* = 3.37, *xyz* = −28, −4, −15) (Bonferroni corrected for SVC FWE‐corrected *P* < 0.01) (Fig. 1). All other ROI analyses were not significant. Adjusting for depression did not change the group difference findings.

### Resting State

**Figure 2:**
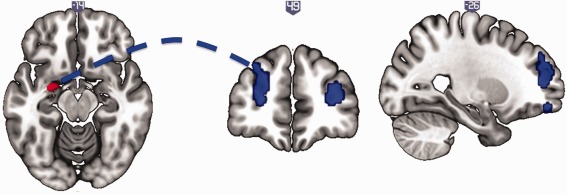
Resting state functional connectivity of the left amygdala. Compulsive sexual behavior is associated with decreased resting state functional connectivity of the left amygdala (seed, left) with bilateral dorsolateral prefrontal cortex (middle and right), relative to healthy volunteers. The image is thresholded at *P* < 0.005 uncorrected for illustration. [Color figure can be viewed at http://wileyonlinelibrary.com]

Based on the structural results, we examined resting state functional connectivity with a seed in the left amygdala. We found reduced connectivity with bilateral DLPFC (Right DLPFC: *P* = 0.012, *Z* = 4.11, *xyz* = 31 42 16; Left DLPFC: *P* = 0.003, *Z* = 3.96, *xyz* = −27 52 23) (Fig. 2). Adjusting for BDI did not alter the significance of the findings (Right DLPFC: *P* = 0.001, *Z* = 4.54, *xyz* = 31 61 23; Left DLPFC: *P* = 0.003, *Z* = 4.26, *xyz* = −29 49 35).

## DISCUSSION

We investigated structural and functional neural differences in individuals with CSB compared with matched HV. CSB subjects had increased left amygdala volume and reduced functional connectivity during rest between the left amygdala and bilateral DLPFC.

The amygdala is implicated in environmental salience processing that directs behavior. The nuclei of the amygdala link previously neutral environmental or internal stimuli with associative representations of affective value, propagating cue‐induced motivational salience [Everitt et al., 2003], as well as processing of emotional control [Cardinal et al., 2002; Gottfried et al., 2003]. The finding of increased amygdala volume is in opposition with several studies on alcohol use disorders [Makris et al., 2008; Wrase et al., 2008], as studies across this type of addiction report decreased amygdala volumes, where volumetric measures have been assessed. A potential explanation for this discrepancy is that long‐term substance use result in long‐lasting neuroplastic changes and toxicity [Kovacic, 2005; Reissner and Kalivas, 2010] that may contribute to the perseveration of drug‐seeking behavior [Gass and Olive, 2008]. Such neurotoxicity may certainly contribute to the widespread atrophy observed in substance addictions [Bartzokis et al., 2000; Carlen et al., 1978; Mechtcheriakov et al., 2007]. Such drug‐related neurotoxicity is likely a highly relevant issue in SUD but less of an issue in behavioral addictions. In a recent CSB study using fMRI, exposure to sexually explicit cues in CSB compared with non‐CSB subjects was associated with activation of the amygdala [Voon et al., 2014]. Whether the difference in amygdala volume is a pre‐existing trait predisposing individuals to CSB or related to excessive exposure remains to be established.

The functioning of the DLPFC is well known to be associated with broad aspects of cognitive control [MacDonald et al., 2000] and working memory [Petrides, 2000]. Our finding of decreased functional connectivity between amygdala and DLPFC converges with the existing literature on the connectivity in these regions. This functional connectivity is important for emotion regulation, which has previously been reported in that reduced connectivity between amygdala and DLPFC in individuals with internet gaming disorder is associated with higher levels of impulsivity [Ko et al., 2015]. Another study measuring the capacity to modulate negative emotional responses through the use of cognitive strategies showed that activity in specific areas of the frontal cortex, including DLPFC, covaried with amygdala activity, and that functional connectivity between these regions was dependent on applying cognitive strategies in the regulation of negative emotion [Banks et al., 2007]. Amygdala and DLPFC connectivity has similarly been associated with unipolar depression [Siegle et al., 2007]. CSB has been associated with depressive and anxiety symptoms and stress may trigger such activities; however, our findings were unrelated to depression scores. The DLPFC was also implicated in a study of male HV in which greater pornography use was associated with lower functional connectivity between the DLPFC and striatum when viewing explicit imagery [Kühn and Gallinat, 2014].

We caution that these findings are preliminary given the small sample size of CSB subjects although notably we compare this group with a large sample size of matched HV. One limitation of the study is the homogeneity of the population. As we did not include subjects with other comorbid psychiatric disorders which might play a mechanistic role, these results should be cautiously extrapolated to CSB subjects with other comorbidities. Furthermore, the observed structural and functional abnormalities among the CSB subjects may be related to pre‐existing traits or may be a result of the effects of CSB, and as such this study cannot make causal inferences about the effects of CSB. Future studies should aim to assess longitudinal measures to determine differences between state and trait tendencies and potential pre‐morbid neural abnormalities in larger sample sizes and with mixed genders.

Our current findings highlight elevated volumes in a region implicated in motivational salience and lower resting state connectivity of prefrontal top‐down regulatory control networks. Disruption of such networks may explain the aberrant behavioral patterns toward environmentally salient reward or enhanced reactivity to salient incentive cues. Although our volumetric findings contrast with those in SUD, these findings may reflect differences as a function of the neurooxic effects of chronic drug exposure. Emerging evidence suggests potential overlaps with an addiction process particularly supporting incentive motivation theories. We have shown that activity in this salience network is then enhanced following exposure to highly salient or preferred sexually explicit cues [Brand et al., 2016; Seok and Sohn, 2015; Voon et al., 2014] along with enhanced attentional bias [Mechelmans et al., 2014] and desire specific to the sexual cue but not generalized sexual desire [Brand et al., 2016; Voon et al., 2014]. Enhanced attention to sexually explicit cues is further associated with preference for sexually conditioned cues thus confirming the relationship between sexual cue conditioning and attentional bias [Banca et al., 2016]. These findings of enhanced activity related to sexually conditioned cues differ from that of the outcome (or the unconditioned stimulus) in which enhanced habituation, possibly consistent with the concept of tolerance, increases the preference for novel sexual stimuli [Banca et al., 2016]. Together these findings help elucidate the underlying neurobiology of CSB leading toward a greater understanding of the disorder and identification of possible therapeutic markers.
